# In Silico Insights on the Pro-Inflammatory Potential of Polycyclic Aromatic Hydrocarbons and the Prospective Anti-Inflammatory Capacity of *Andrographis paniculata* Phytocompounds

**DOI:** 10.3390/ijerph19148588

**Published:** 2022-07-14

**Authors:** Trixia Julaton, Aibelou Taclendo, Glenn Oyong, Ofelia Rempillo, Maria Cecilia Galvez, Edgar Vallar

**Affiliations:** 1 Environment and RemoTe Sensing Research (EARTH) Laboratory, Department of Physics, College of Science, De La Salle University Manila, 2401 Taft Avenue, Manila 0922, Philippines; trixia_julaton@dlsu.edu.ph (T.J.); aibelou_taclendo@dlsu.edu.ph (A.T.); ofelia.rempillo@dlsu.edu.ph (O.R.); maria.cecilia.galvez@dlsu.edu.ph (M.C.G.); 2 Molecular Science Unit Laboratory, Center for Natural Sciences and Ecological Research, De La Salle University, 2401 Taft Avenue, Manila 0922, Philippines; glenn.oyong@dlsu.edu.ph

**Keywords:** inflammation, polycyclic aromatic hydrocarbons, TLR-4, *Andrographis paniculata*, NF-κB p50, molecular docking, molecular dynamics simulation

## Abstract

Inflammation linked to various diseases is the biological response to certain stimuli. The pro-inflammatory potential of Polycyclic Aromatic Hydrocarbons (PAHs) as potential inducers of inflammation bound to the Toll-like Receptor 4 (TLR4) and the anti-inflammatory capacity of *A. paniculata* (AP) phytocompounds as prospective inhibitors of the Nuclear Factor Kappa B (NF-κB) p50 transcription factor are investigated via in silico techniques. The molecular docking of the PAHs and AP phytocompounds is performed in AutoDock Vina by calculating their binding energies. The molecular dynamics simulations (MDS) of the apo and ligand-bound complex of the top binding ligands were performed in CABS-flex. The agonists, which included the PAHs indeno(1,2,3-cd)pyrene (IP), and dibenz(a,h)anthracene (DahA), had the highest binding energies of −10 kcal/mol and −9.2 kcal/mol, respectively. The most stable antagonists in the binding site with binding energies to the NF-κB p50 were the AP phytocompounds with −5.6 kcal/mol for ergosterol peroxide and −5.3 kcal/mol for 14-deoxy-14,15-dehydroandrographolide. The MDS of the apo human TLR4 and PAH-bound TLR4, and the apo p50 and the AP phytocompound-bound NF-κB p50 showed minimal fluctuations. These results reveal that IP and DahA are significant inducers of inflammation, whereas ergosterol peroxide and 14-deoxy-14,15-dehydroandrographolide are inhibitors of the NF-κB pathway. Furthermore, the study theorizes that any inflammatory activity induced by PAH can be potentially inhibited by *A. paniculata* phytocompounds.

## 1. Introduction

Polycyclic aromatic hydrocarbons (PAHs) are widespread air pollutants in the environment that exacerbate adverse health effects in metabolic disorders by inducing inflammation and oxidative stress [1]. These ubiquitous environmental pollutants are produced during the incomplete combustion of organic materials such as coal, oil, or wood. The Philippines is a large consumer of coal due to its use in power generation. These conditions make people more prone to inflammatory disorders due to repeated PAH exposure. 

Santiago and Cayetano, in 2007 [2], reported that PAH concentrations found in the residential areas are influenced by their proximity to sources of combustion and emission. Further analysis showed that the highest and lowest concentration of PAHs recorded in the Philippines were higher than the average range of PAH concentrations reported in London and Manchester. Moreover, Zhang et al. reported that PAHs found in the surface water of the Philippine Sea are mostly 3,4-Ring PAHs that come from coal combustion [3]. Despite the ban on new power plants in 2017, the Philippine energy department still allowed 22 previously approved power plants which are expected to increase the country’s coal account by 53% by 2030 [4]. Thus, this increases the risk of onset and progression of chronic inflammatory diseases in the Philippines. 

Acute inflammation is an ideal, rapid, and short-lived inflammatory response of the body to eliminate pathological stimuli and promote therapeutic activities. However, uncontrolled inflammation due to specific social, psychological, environmental, and biological factors marks the onset of low-grade, non-infective, systemic chronic inflammation [5]. In the Philippines, these factors are prominent; hence, the overall quality of life of the population is affected. 

Non-steroidal anti-inflammatory drugs (NSAIDs), corticosteroids, and anti-cytokine biologics are widely used as anti-inflammatory medications for acute and chronic inflammation. However, the use of these drugs is associated with corticosteroid resistance, drug dose-limiting side effects, and inconvenient routes of administration [6]. For rheumatoid arthritis, andrographidine C of *A. paniculata* has been reported to be a potent anti-inflammatory drug [7]. The phytocompound, associated with fewer or minor adverse effects, may be utilized over disease-modifying anti-rheumatic drugs (DMARDs) and non-steroidal anti-inflammatory drugs (NSAIDs). Traditional medicine is harnessed in nearly two-thirds of developing country populations to meet primary healthcare needs [8]. Thus, one plant species that could provide treatments is *Andrographis paniculata* (Burm. f.) Nees, commonly called “Sinta”, which contains several phytocompounds that have been identified as having potent anti-inflammatory, antitumor, antioxidative, antimicrobial, and cardioprotective pharmacological properties [9]. *A. paniculata* is cultivated and used as traditional medicine in many countries, including India, China, Hongkong, Pakistan, Bangladesh, Malaysia, the Philippines, Indonesia, and Thailand [10].

Andrographolide, the leaf’s most abundant phytocompound, can be easily isolated from the crude plant extract as a crystalline solid [11]. In various inflammatory disease models, the inhibition of NF-κB signaling pathways by andrographolide has been observed [6]. In an LPS-induced inflammatory response (TNF-α secretion and IL-1α/β expression) through the induction of NF-κB and C/EBP transcriptional activity and phosphorylation of p38, JNK, and ERK MAPKs, ergosterol peroxide suppressed the response by inhibition of the activations in RAW264.7 macrophage-like cells [12]. Ergosterol peroxide, a C-28-sterol, is a component of *A. paniculata* and many medicinal mushrooms. This phytocompound has been identified as having anti-rheumatoid, antimicrobial, cytotoxic, and immunosuppressive biological activities [7,13]. A diterpenoid bioactive compound in *A. paniculata*, 14-deoxy-14,15-dehydroandrographolide, is reported as anti-inflammatory [11,14]. A previous in vitro study confirmed that 14-deoxy-14,15-dehydroandrographolide, one of the ethyl acetate extracts of *A. paniculata*, exerted the strongest inhibitory effects on NF-κB-dependent transactivation, an acute inflammation, in RAW 264.7 cells [15]. For an in vivo study, extracts from *Andrographis paniculata* can significantly suppress the infiltrations of neutrophils and lymphocytes and reduce the excessive production of cytokines and chemokines in rats in a dose-dependent manner [16].

Several studies have examined the association and mechanisms by which PAHs utilize TLR4 in eliciting cellular responses such as inflammation [17,18,19]. Furthermore, a review report mentioned the ability of *Andrographis paniculata* to regulate the expression of cytochrome P450 family 1 subfamily A (CYP1A1) mRNA expression, a gene found to have significant influence in inducing cellular responses caused by chemical carcinogens such as PAHs [20]. An in silico screening could identify which phytocompound in *A. paniculata*, alone, or in combination, can block the PAH-induced activation of the NF-κB pathway and could serve as a preliminary study for a future in vitro investigation. Therefore, this study aims to test the pro-inflammatory potential of selected PAHs in activating the NF-κB pathway, investigate the anti-inflammatory effect of AP phytocompounds, and illuminate a potential mechanism. 

## 2. Materials and Methods

To investigate the anti-inflammatory effect of AP phytocompounds on PAH-induced inflammation, an in silico approach was performed as a preliminary study to an in vitro study at little to no cost. This section discusses the methods of the retrieval and preparation of all ligand and protein structures, validation of protein stability, molecular docking, and the molecular dynamic simulation performed in the study. Three-dimensional conformational structures of seven PAHs and nine bioactive phytocompounds from *A. paniculata* were retrieved from the PubChem database. These ligand structures were analyzed in terms of their respective proteins (human TLR4 and NF-κB p50 homodimer) using AutoDock Vina 1.2.0 and CABS-flex 2.0. Considering that the study utilized a considerable number of compounds, in silico was an appropriate approach to identify which AP phytocompounds can demonstrate the most comparable anti-inflammatory activity to clinically approved NF-κB inhibitors. It could also reduce expensive laboratory work and the time required to predict AP’s anti-inflammatory potential against PAH-induced activation of NF-κB proteins in the cytoplasm.

**Figure 1:**
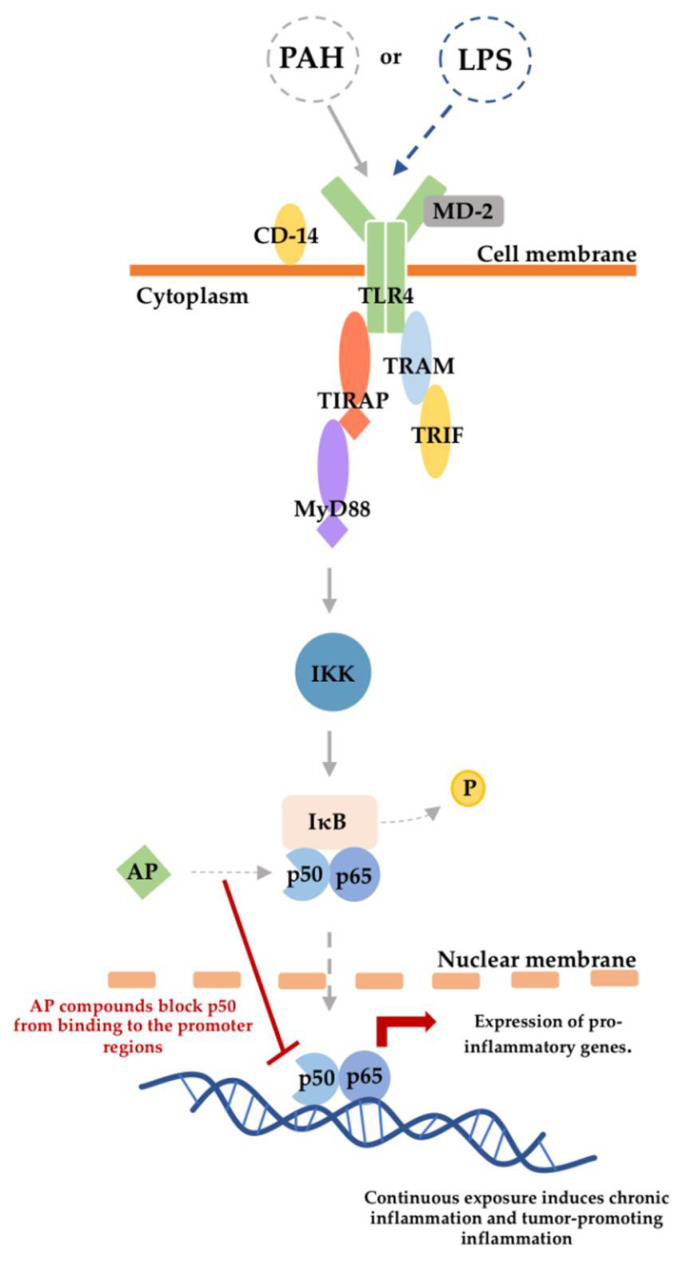
Mechanistic pathway of PAH-induced inflammation via the TLR4 pathway and AP inflammatory inhibition via the canonical NF-κB pathway.

PAHs, components of particulate matter, stimulate the TLR4 and lead to the activation of the pro-inflammatory NF-κB pathway. Figure 1 shows that a cascade of pro-inflammatory cytokines is initiated when a PAH or an LPS binds to the human Toll-like receptor 4 (TLR4). The cascade of pro-inflammatory cytokines leads to the activation of the IkappaB kinase (IKK), an essential protein complex that regulates inflammation in both the canonical and non-canonical NF-κB pathway [21]. IKK initiates the phosphorylation of IκB to free the p50/p65 heterodimer [21,22]. Consequently, the free p50/p65 heterodimer initiates the transcription of pro-inflammatory genes [22,23]. The introduction of AP to the binding site of the p50 heterodimer prevents the p50 dimer from binding to the promoter region of the target gene, thus, inhibiting the expression of pro-inflammatory genes.

### 2.1. Retrieval and Preparation of PAH Ligand Structures

**Figure 2:**
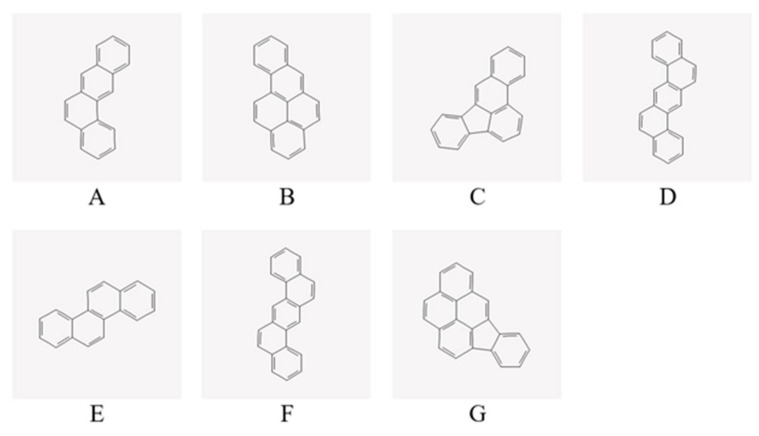
Molecular structures of the seven probable human carcinogens categorized by the U.S. Environmental Protection Agency: (**A**) benz(a)anthracene, (**B**) benzo(a)pyrene, (**C**) benzo(b)fluoranthene, (**D**) benzo(k)fluoranthene, (**E**) chrysene, (**F**) dibenz(ah)anthracene, and (**G**) indeno(1,2,3-cd)pyrene.

The 3D conformer of seven different polycyclic aromatic hydrocarbons (PAHs) agonist ligands of interest (benz(a)anthracene, benzo(a)pyrene, benzo(b)fluoranthene, benzo(k)fluoranthene, chrysene, dibenz(a,h)anthracene, and indeno(1,2,3-cd)pyrene) were retrieved from the database of PubChem in structure data file (SDF) format [9]. To carry out the molecular docking study, these PAHs were selected based on their classification as probable human carcinogens by the U.S. Environmental Protection Agency [10]. The selected ligands were entered into the homepage’s search bar and the SDF files of each compound were converted into Protein Data Bank, Partial Charge (Q), Atom Type (T) (PDBQT) file format using Open Babel graphical user interfaces (GUI) version 2.4.1 [24]. The two-dimensional conformations of the seven mentioned PAHs are found in Figure 2. 

### 2.2. Retrieval and Preparation of AP Ligand Structures

**Figure 3:**
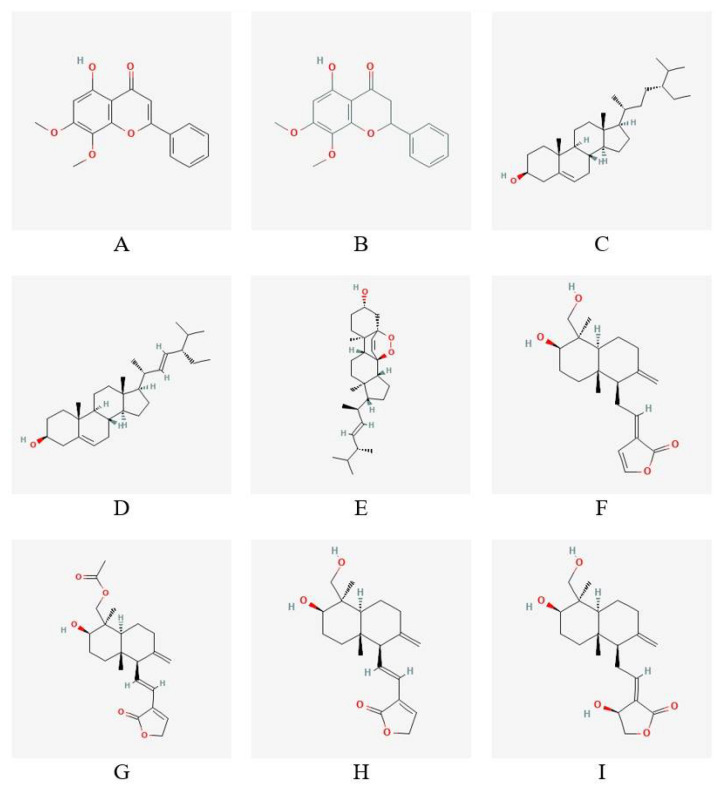
Molecular structures of the compounds from *Andrographis paniculata* that inhibit NF-κB transactivation: (**A**) 5-hydroxy-7,8-dimethoxyflavone, (**B**) 5-hydroxy-7,8dimethoxyflavanone, (**C**) β-itosterol, (**D**) stigmasterol, (**E**) ergosterol peroxide, (**F**) 14-deoxy14,15-dehydroandrographolide, (**G**) 19-O-acetyl-14-deoxy-11,12-didehydroandrographolide, (**H**) 14-deoxy-11,12-didehydroandrographolide, and (**I**) andrographolide.

The nine (9) candidate anti-inflammatory compounds (antagonists) are as follows: 5-hydroxy-7,8-dimethoxyflavone, 5-hydroxy-7,8-dimethoxyflavanone, β-sitosterol, stigmasterol, ergosterol peroxide, 14-deoxy-14,15-dehydroandrographolide, 19-O-acetyl-14-deoxy-11,12-didehydroandrographolide, 14-deoxy-11,12-didehydroandrographolide, and andrographolide. The 3D conformers of these compunds were obtained from PubChem in SDF format and converted into PDBQT file format. The selection included the active compounds from bioactivity-guided chromatographic fractionation of the pure compounds in the ethyl acetate fraction of *Andrographis paniculata* [15]. They possessed anti-inflammatory activities that inhibit the transcriptional activity of NF-κB in LPS/IFN-γ stimulated RAW 264.7 macrophages. The isolated andrographolide has shown less inhibitory activity but exerted greater inhibitory activity in derived compounds formed by hydrogenation, oxidation, or acetylation [15]. The two-dimensional conformations of the compounds from *Andrographis paniculata* are shown in Figure 3.

### 2.3. Retrieval of Protein Structures and Validation

The 3D crystal structure of human Toll-like receptor 4 (TLR4) polymorphic variant complexed with lipopolysaccharide (LPS) was obtained from the RCSB-PDB database in Protein Data Bank (PDB) format file and is associated with the macrophage cells (PDB ID 4G8A) [25]. The protein data bank is a publicly available central archive of all experimentally determined protein structure data and biological compounds which have three-dimensional structural information. The protein database ID of the protein structure of interest was accessed using the website’s search function. For the investigation of the anti-inflammatory compounds (antagonists), the 3D structure of the nuclear factor kappa-B p50 homodimer bound to a kappa B site in PDB format was obtained in RCSB-PDB (PDB ID: 1NFK).

In the preparation of human TLR4, the binding sites of LPS bound to the protein dimer were found via BIOVIA Discovery Studio Visualizer 2021. The grid parameters for the localization of the binding site in the XYZ sphere are set as follows: 15 Å radius with center coordinates, −22.061059; −17.735272; −17.924471. Exhaustiveness with the value of 8 is the default. The bond between the ligand and protein was visualized using the ‘show ligand binding interaction’ command. Moreover, the bonds between ligands and the amino acids and types of bonds in a two-dimensional view were visualized using the ‘show 2D diagrams’ command. 

The extra subunits, water molecules, and other heteroatoms were removed from the structure. The LPS bound to the TLR4 was also deleted for the seven PAH compounds each added later in AutoDock Tools 4.2. AutoDock Tools is a grid-based docking algorithm used to add polar hydrogen atoms and Kollman charges in the study [26,27]. Visualization on the interaction between ligands and amino acids is not visible in AutoDock; hence, BIOVIA Discovery Studio Visualizer is needed and the diagrams are saved.

The preparation of the p50 subunit was performed following the same steps used in the preparation of TLR4. The grid parameters for the binding site in p50 in the XYZ sphere are: 15 Å radius with center coordinates, −2.330858; 14.689547; 20.134008, and default 8 units for the exhaustiveness. The extra subunits, water molecules, and other heteroatoms were removed from the structure of the p50 subunit. The DNA attached from the p50 model was also removed for the 9 AP compounds.

Zlab, Verify3D, and ERRAT were performed for the detection of errors in the TLR4 and the p50 binding site models. The PDB file of the prepared proteins were uploaded, and the Ramachandran plot was run and obtained using Zlab. The Zlab Ramachandran plot server is a server that validates the refined prepared protein model by estimating the structural stability. VERIFY3D and ERRAT programs were both performed in the SAVES v6.0—Structure Validation Server by uploading the PDB file of the prepared TLR4 protein. VERIFY3D determines the compatibility of the 3D structures and the primary sequence in the refined protein model were analyzed, respectively. ERRAT is a web server program used to verify protein structures determined by crystallography and its plot shows error values for residues. Calculated error values falling below the 95% rejection limit would imply that the proteins run in the ERRAT web server are stable.

### 2.4. Molecular Docking

The molecular docking model was employed to ascertain the theoretical binding interactions of (1) benz(a)anthracene, (2) benzo(a)pyrene, (3) benzo(b)fluoranthene, (4) benzo(k)fluoranthene, (5) chrysene, (6) dibenz(ah)anthracene, and (7) indeno(1,2,3-cd)pyrene with the prepared TLR4 protein. AutoDock Vina, a molecular docking algorithm employing knowledge-based potentials and empirical scoring functions, was run using a command prompt, and the top nine scores were shown. A more negative binding energy (kcal/mol) is preferable. This depends on the score and probability of binding to the protein’s active-site pocket. The RMSD (lower bound) and RMSD (upper bound) of the docking poses are also generated and evaluated. The TLR4-PAH compound complex with the lowest binding energy is used as the most favored confirmation to explain the interaction. The molecular interaction of the protein with their respective polycyclic aromatic hydrocarbon pollutants was each studied using AutoDock Vina through parameters encompassing intermolecular energy (hydrogen bonding energy, Van der Waals energy, electrostatic energy, etc.), internal energy and torsional energy. The molecular docking was repeated for each ligand. The strong binding affinities with the protein and their association with Van der Waal and hydrophobic interactions were examined. The compounds with the highest binding affinities were identified based on prominent interactions in the results. The present study was conducted in silico, and thus ethical issues were not involved. 

In the in silico molecular docking study, docking of the anti-inflammatory compounds, (1) 5-hydroxy-7,8-dimethoxyflavone, (2) 5-hydroxy-7,8-dimethoxyflavanone, (3) β-sitosterol, (4) stigmasterol, (5) ergosterol peroxide, (6) 14-deoxy-14,15-dehydroandrographolide, (7) 19-O-acetyl-14-deoxy-11,12-didehydroandrographolide, (8) 14-deoxy-11,12-didehydroandrographolide, and (9) andrographolide, with the prepared NF-κB p50 subunit proteins was investigated. The same steps as with the TLR4 molecular docking were followed for the p50 protein. As a proposed solution to the inflammation caused by PAHs, the p50-AP compound complex with the lowest binding energy (highest binding affinity) was used as the most favored conformation to explain the theoretical binding interactions. The same variables studied in the PAHs were studied in the respective *Andrographis paniculata* compounds. The phytocompounds with the highest binding affinities were investigated due to prominent interactions in the molecular docking results.

The binding energies of LPS and dexamethasone were used as a reference standard (positive control) for the comparison with the ligands with the highest binding affinities. A ligand with a lower binding energy than the positive controls was shown to be a potent ligand. For reference, the binding energies are −4.1 kcal/mol for LPS and −5.4 kcal/mol for dexamethasone.

### 2.5. Molecular Dynamics Simulation (MDS)

Molecular dynamics simulations are performed to validate the stability of the protein-ligand complexes and to examine the conformational changes after interactions. PDB files of the unbound TLR4, TLR4 bound with its top binding ligands, unbound NF-κB p50 transcription factor, and NF-κB p50 bound with its top binding ligands were each submitted to the CABS-flex 2.0 web server, applying the default parameters. CABS-flex 2.0 generates models which represent the changes in its conformation structure during 10 nanoseconds. The fluctuation plots on each chain represent the changes in the number of amino acid residues against the RMSF in Å units. In the human TLR4, there are four protein chains (i.e., A, B, C and D), whereas in NF-κB p50, there are two protein chains (i.e., A and B). The raw data of the unbound and bound structures per chain were then used to determine the statistical differences between the models (unbound and bound). A two-sample paired t-test with a significance level (α) of 0.05 was used to determine the *p*-value in Microsoft Excel Version 16.56. For the visualization of the models and their superimposition, the differences in the unbound protein at 0 ns to the bound protein at 10 ns, the superimposition of all the unbound model structures from 0 to 10 ns, and all superimposed models of the bounded structures from 0 to 10 ns, were visualized further in Discovery Studio Visualizer.

## 3. Results

### 3.1. Structural Stability of Prepared Proteins

**Figure 4:**
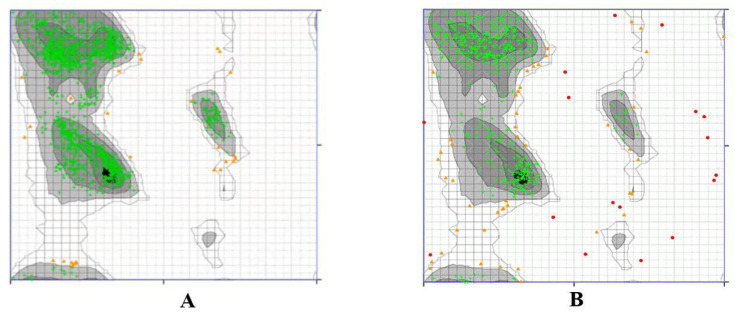
Ramachandran plot by Zlab of the defined binding site in (**A**) TLR4 and (**B**) p50. The green crosses represent the amino acid residues and are indicative of highly preferred observations; the brown triangles denote preferred observations, and the red circles denote outliers.

A Ramachandran plot was created based on the van der Waal radius of the side chains. As shown in Figure 4, the Ramachandran plots of the TLR4 (Figure 4A) and p50 (Figure 4B) protein structures show the allowed and rejected dihedral angles, psi (ψ) and phi (φ), that form an amino acid. In the defined binding site of TLR4, 98.081% of amino acids, either in an alpha helix or beta-sheet, were perfectly aligned, thus indicating that the prepared TLR4 protein was acceptable. Furthermore, there were no outliers observed in the TLR4 protein plot. In the defined binding site of p50, 88.268% of amino acids, either in an alpha helix or beta-sheet, were perfectly aligned, thus indicating that the p50 protein was acceptable. In the p50 protein plot, there were 3.166% observed outliers.

**Figure 5:**
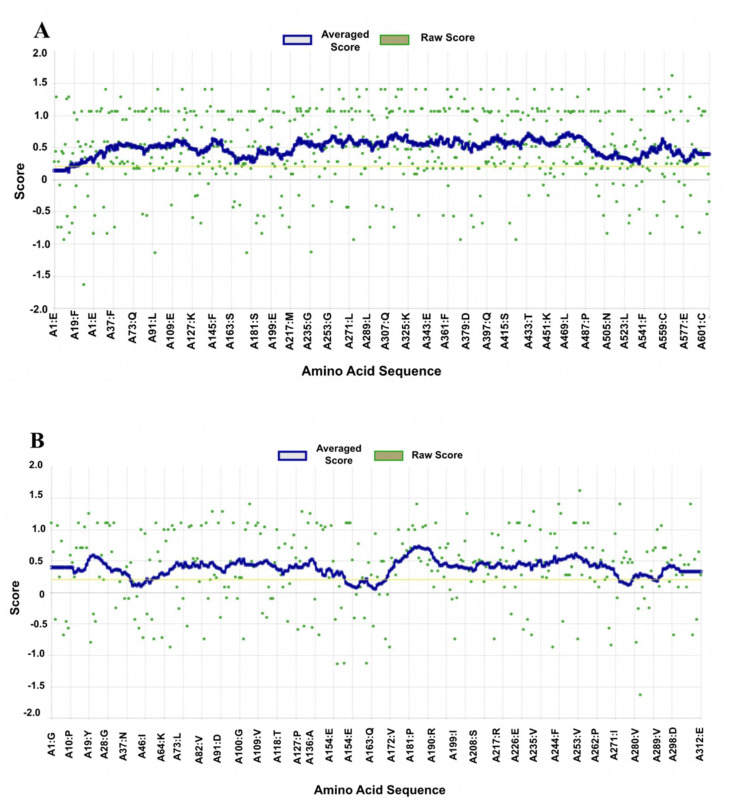
VERIFY3D plots showing at least 80% of the amino acids scored ≥ 0.2 in the 3D/1D profile: (**A**) TLR4 protein and (**B**) p50 protein.

Verify3D showed that 96.57% of the residues in the TLR4 protein had an average 3D-1D score ≥ 0.2, which met the 80% score requirement. Similarly, the Verify3D reliability testing of the p50 protein showed that 94.07% of the residues had an average 3D-1D score ≥ 0.2 and, therefore, also met the requirement that at least 80% of amino acids have scores ≥ 0.2. Thus, as shown in Figure 5, the TLR4 and p50 proteins are of high quality.

**Figure 6:**
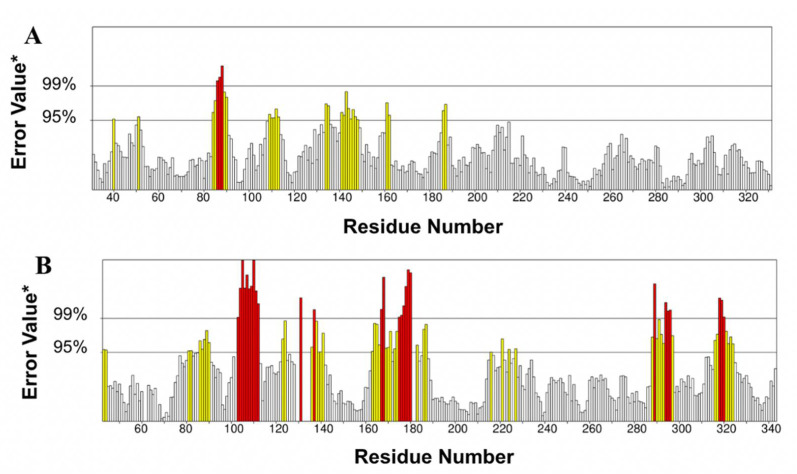
ERRAT plots showing an overall quality factor of 89.1799% in (**A**) TLR4 and 75.8092% in (**B**) p50. * On the error axis, the two line are drawn to indicate the confidence with which it is possible to reject regions that exceed that error value.

There were 89.1799% and 75.8092% of proteins expressed in the ERRAT plot of defined binding sites in TLR4 and p50, respectively. Both calculated error percentages fall below the 95% error rejection limit, and prepared proteins that passed are shown in Figure 6. After the stability of the prepared proteins was verified, each prepared protein was tested for molecular docking in AutoDock Vina v.1.2.0 [28].

### 3.2. PAHs and Human TLR4 Interaction

#### 3.2.1. Analysis of Docking Scores and Amino Acid Interactions

In this study, in silico molecular docking technique was applied to predict the ligands’ conformation, position, and orientation within the human TLR4 binding site. This technique also makes use of scoring functions to determine the efficiency of protein–ligand interactions. Protein–ligand interaction efficiency scores are expressed as binding affinity in MGL Tools such as AutoDock Vina. The results showed that LPS has a binding affinity of −4.1 kcal/mol. Thus, polycyclic aromatic hydrocarbon compounds that have binding energies lower than −4.1 kcal/mol are deemed acceptable sources of inflammation. After the molecular docking simulation process using AutoDock Vina, the conformation with the lowest binding energy was evaluated using Discovery Studio (Biovia).

The amino acids of human TLR4 interacting with LPS are listed in Table 1. Potential sources of inflammation during the interaction between the seven (7) PAHs and the amino acid residues of TLR4 were observed to be Leu61, Ile32, Ile52, Val48, and Phe119. These five amino acid residues of TLR4 may be potential sources of inflammation due to common amino acid interactions observed in the LPS-TLR4 and the PAH-TLR4 complexes.

Table 2 shows the amino acid interactions between the ligands with the highest binding affinity and lowest binding energy. The other two ligands, BkF and BaP, are provided as examples in Section 4.

A summary of the amino acid interactions between the ligands with the highest binding affinities and TLR4 can be found in Table 2. Based on this, a total of five interacting amino acid residues in LPS, IP, and DahA are found to be in common, namely Val48, Ile52, Ile32, Leu61, and Phe119.

In AutoDock Vina, the docking calculations for the binding affinities of each of the seven polycyclic hydrocarbon compounds were based on the ligand’s flexibility while keeping the human TLR-4 rigid. The seven polycyclic hydrocarbon compounds were chosen for their classification as possible human carcinogens by the U.S. Environmental Protection Agency (EPA). The results from the molecular docking calculations, which range from −10 kcal/mol to −8.1 kcal/mol, are shown in decreasing order of priority (from the most negative binding affinity being the highest to the least negative being the lowest) in Table 3.

The results show that all ligands had lower binding energies than LPS, which had a binding energy of −4.2 kcal/mol. Furthermore, the root mean square deviation (RMSD) for the binding energies of the LPS and the seven PAHs was 0.00 angstrom (Å), which means efficacious docking as this parameter validates the accuracy of the molecular docking. At 0 Å, the divergence between the coordinates of the crystallized complex of the simulated complex is determined. Hence, an RMSD value of 0 Å suggests the best docking pose in the binding site (i.e., lower binding energy, higher ranking, stronger binding affinity).

Based on Table 3, indeno(1,2,3-cd)pyrene (IP) and dibenz(a,h)anthracene (DahA) have the most negative binding energies among all the seven PAHs, with binding energies of −10 kcal/mol and −9.2 kcal/mol, respectively. The toxic equivalent factors and carcinogenic classification of the ligands listed in Table 3 are based on the EPA of Tasmania [29] and the U.S. EPA, respectively. Furthermore, the toxic equivalent factor is based on the toxicity of benzo(a)pyrene (BaP), as this is considered the most toxic and carcinogenic PAH. Therefore, ligands with TEF = 1 would have toxicity levels equal to the toxicity of BaP.

**Figure 7:**
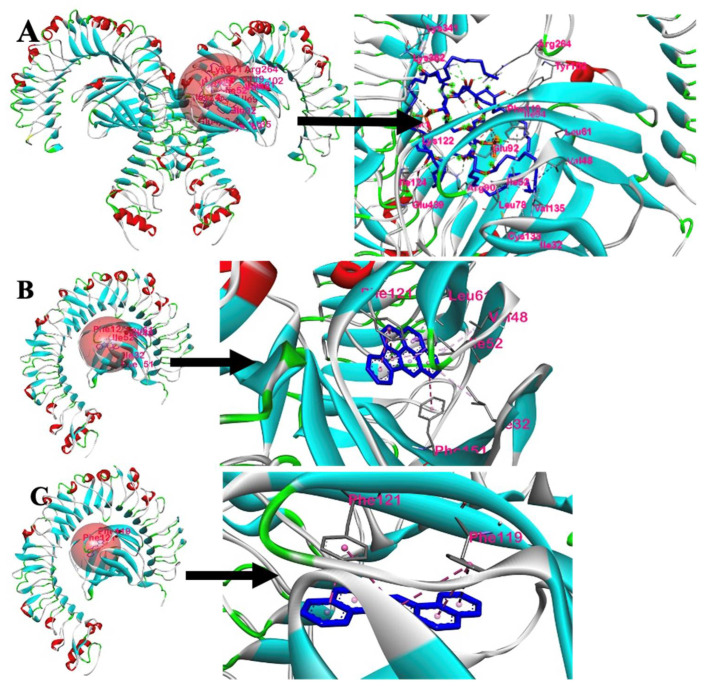
Receptor–ligand interactions: (**A**) LPS with TLR4, (**B**) Indeno(1,2,3-cd)pyrene with TLR4, and (**C**) Dibenz(a,h)anthracene with TLR4.

The interaction of the PAH compounds with the human TLR4 was visualized in BIOVIA Discovery Studio Visualizer. In Figure 7, the binding site locations for LPS, IP, and DahA on TLR4 are defined by the red sphere. Furthermore, the amino acid residues interacting with LPS, IP, and DahA are closely shown on the right side of their respective figures. Looking at Figure 7B,C, only chains B and D of the TLR4 protein are shown since the amino acid interactions between the ligands of interest (DahA and IP) and the amino acids of TLR4 only occurred in chain D of TLR4. Chain B of TLR4 was shown to visualize the binding site better. In contrast, the binding pose of LPS show chains A, B, C, and D of TLR4 for better visualization since amino acids interaction between LPS and TLR4 occurs in chains A, B, and D, as shown in Figure 7A.

**Figure 8:**
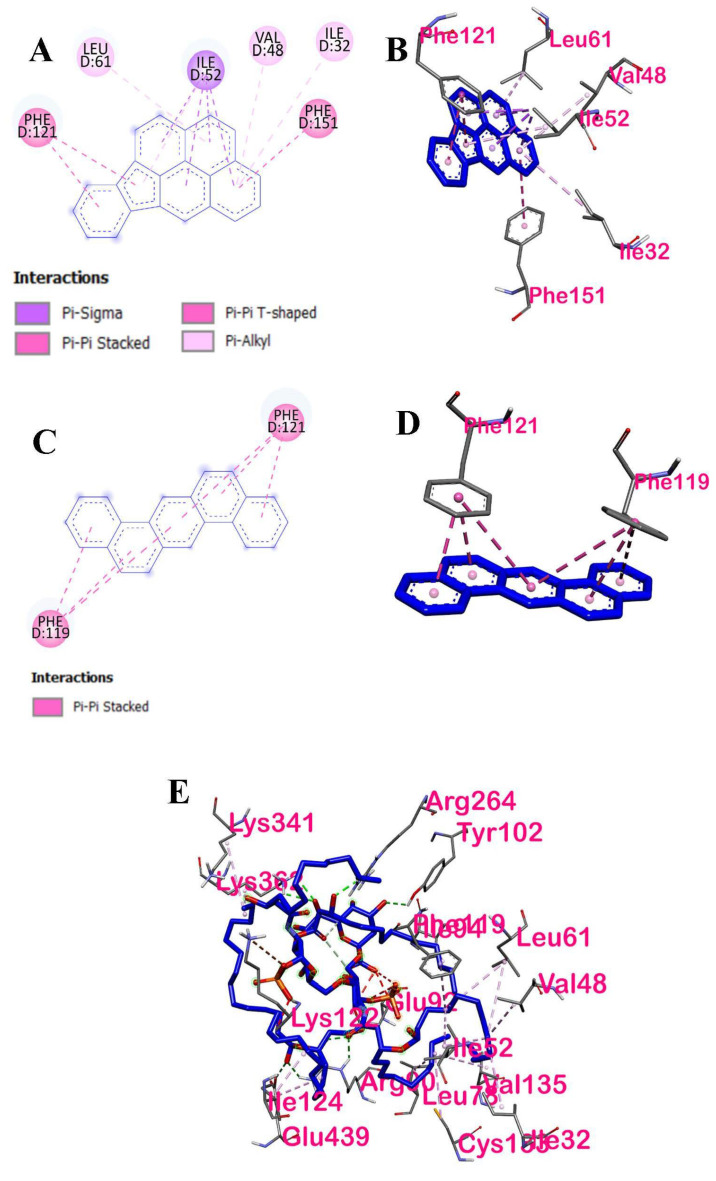
The two- and three-dimensional interactions of LPS, DahA and IP with TLR4: (**A**) 2D interaction of Indeo(1,2,3-cd)pyrene and TLR4; (**B**) 3D interaction of Indeo(1,2,3-cd)pyrene and TLR4; (**C**) 2D interaction of Dibenz(a,h)anthracene and TLR4; (**D**) 3D interaction of Dibenz(a,h)anthracene and TLR4; and (**E**) 3D interaction of LPS and TLR4.

Figure 8 shows the 2D and 3D interactions between LPS and the ligands with the highest binding affinities with TLR4. Based on the 2D and 3D amino acid interactions, six amino acids of the human TLR4 interacted with IP, two amino acids for DahA, and eighteen amino acids for LPS. A 2D interaction of LPS could not be configured due to the limitations of the molecular docking tool used.

#### 3.2.2. Intermolecular Forces of Attraction

**Figure 9:**
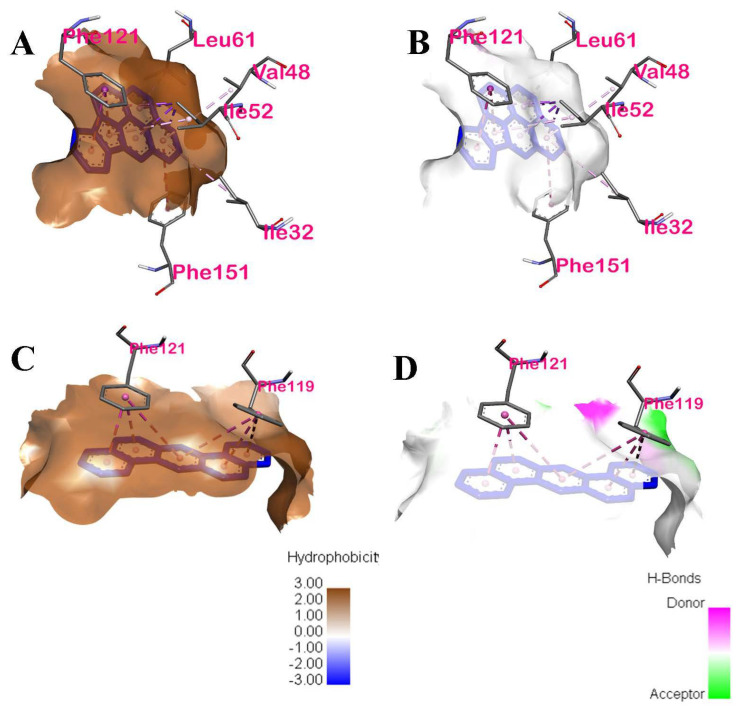
Intermolecular forces of attractions between DahA−TLR4 and IP−TLR4: (**A**) hydrophobic interaction of TLR4 amino acids with Indeno(1,2,3-cd)pyrene; (**B**) H−bonding of TLR4 amino acids with Indeno(1,2,3-cd)pyrene; (**C**) hydrophobic interaction of TLR4 amino acids with Dibenz(a,h)anthracene; (**D**) H-bonding of TLR4 with Dibenz(a,h)anthracene.

The hydrophobic interactions between the ligands, IP and DahA, and the active site residues of TLR4 are shown in Figure 9A,C, respectively. The hydrophobic distribution of DahA and Ip towards their respective binding sites was projected onto the surface of TLR4. It can be observed from the hydrophobicity profiles of the ligands of interest that the interaction of the active pocket amino acids and the ligands was mainly hydrophobic. The hydrophobic interactions of IP and the active site residues found in chain D of TLR4 were pi-sigma, pi-pi stacking, pi-pi T-shaped, pi-alkyl-pi-alkyl, and pi-alkyl for Ile52, Phe121, Phe151, Leu61, Ile32, and Val48 amino acids, respectively. Similarly, the hydrophobic interactions of DahA and the active site residues, Phe121 and Phe119, in the chain D of TLR4, were governed by hydrophobic interactions via pi-pi stacking. Furthermore, the hydrogen bond properties of the binding pocket in IP (Figure 9B) showed that Ile52, Phe121, Phe151, Leu61, Ile32, and Val48 were neither H-bond donors nor acceptors of IP. Similarly, the hydrogen bond properties of the binding pocket in DahA (Figure 9D) showed that Phe121 and Phe119 were neither H-bond donors nor acceptors of DahA. Ile52, Phe121, Phe151, Leu61, Ile32, Val48, and Phe119 cannot act as proton donors or acceptors of IP and DahA since the interactions of these two ligands with the mentioned amino acids of TLR4 were strictly hydrophobic.

Additionally, the distance of interaction between the ligands IP and DahA and the amino acids of TLR4 are shown in Table 4. Chain D Ile52 of TLR4 interacts hydrophobically with IP by pi-sigma at three (3) locations at distances of 3.3891 Å, 3.89425 Å, and 3.44148 Å, and by pi-alkyl at one (1) location of the ligand at a distance of 4.62034 Å; chain D Phe121 of TLR4 interacts hydrophobically with IP by pi-pi stacking at two (2) locations at distances of 4.14965 Å, and 4.64929 Å; chain D Phe151 of TLR4 interacts hydrophobically with IP by pi-pi T-shaped at one (1) location by a distance of 5.84498 Å; chain D Leu61 of TLR4 interacts hydrophobically with IP by pi-alkyl at one (1) location by a distance of 5.18222 Å; chain D Ile32 interacts hydrophobically with IP by pi-alkyl at one (1) location by a distance of 5.47123 Å; and chain D Val48 interacts hydrophobically with IP by pi-alkyl at one (1) location by a distance of 5.16885 Å. On the other hand, chain D Phe119 of TLR4 interacts hydrophobically with DahA by pi-pi stacking at three (3) locations at distances of 5.52106 Å, 4.47965 Å, and 5.42439 Å, and chain D Phe121 also interacts hydrophobically with DahA by pi-pi stacking at three (3) locations at distances of 5.11694 Å, 3.86586 Å, and 3.74824 Å.

#### 3.2.3. Ramachandran Plot

**Figure 10:**
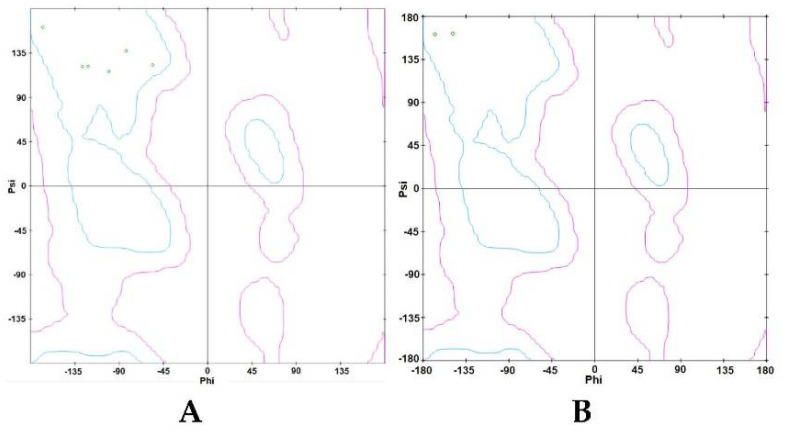
Ramachandran plot of ligands with the highest binding affinity: (**A**) Ramachandran plot of Indeno(1,2,3-cd)pyrene; (**B**) Ramachandran plot of Dibenz(a,h)anthracene. Small green circular portions of the Ramachandran plot represent the amino acid residues.

The Ramachandran plot of the ligands with the highest binding affinities is shown in Figure 10. As shown in the Ramachandran plot of IP (Figure 10A), Ile52, Phe121, Phe151, Leu61, Ile32, and Val48 were all within the acceptable or favorable regions. Conformations of Val48, Ile32, Leu61, Phe121, and Ile52 do not experience steric hindrances. Additionally, all six (6) chain D amino acid residues of TLR4 have β-pleated sheet structures. In the Ramachandran plot of DahA (Figure 10B), chain D amino acids of TLR4, namely Phe119 and Phe121, were also both within the favorable regions. Phe119 and Phe121 both have β-pleated sheet structures and have approximate phi and psi angles that would allow them to come a little closer together. Furthermore, secondary structures such as β-pleated sheets are local structures that are stabilized by hydrogen bonds.

#### 3.2.4. Molecular Dynamics Simulation

**Figure 11:**
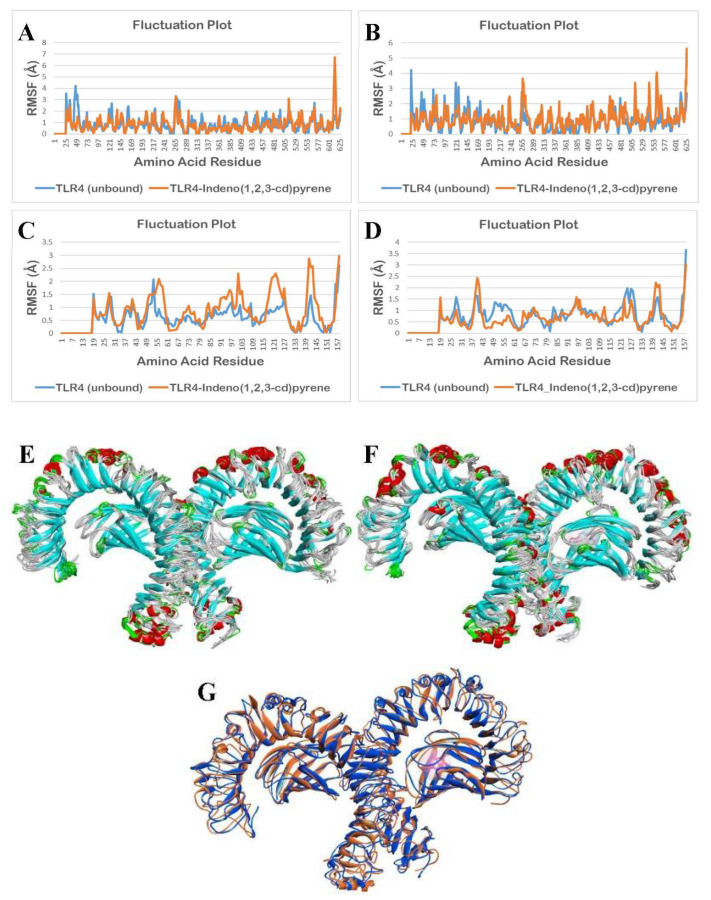
Molecular dynamics simulation of unbound and IP bound TLR4 structures: (**A**) fluctuation plot of the number of amino acid residues of unbound (blue) and bound structure (orange) of TLR4 chain A, (**B**) fluctuation plot of the number of amino acid residues of unbound (blue) and bound structure (orange) of TLR4 chain B, (**C**) fluctuation plot of the number of amino acid residues of unbound (blue) and bound structure (orange) of TLR4 chain C, (**D**) fluctuation plot of the number of amino acid residues of unbound (blue) and bound structure (orange) of TLR4 chain D, (**E**) superimposition of unbound TLR4 structures from 0 ns to 10 ns, (**F**) superimposition of IP bound TLR4 structures from 0 ns to 10 ns, and (**G**) superimposition of unbound TLR4 (blue) at 0 ns and IP (pink) bound TLR4 (orange) at 10 ns.

The molecular dynamics simulation (MDS) was performed using the CABS-flex 2.0 web server, applying the default parameters. Fluctuations in the amino acid residues in the unbound (apo/ligand-free) TLR4 and bound structure of TLR4 with indeno(1,2,3-cd)pyrene (IP) during a 10 ns molecular dynamics simulation are shown in Figure 11.

As shown in Figure 11A,D, it can be observed that there are minimal fluctuations in the TLR4-apo and TLR4-IP structures. A statistical analysis, using a two-tailed t-test at a 0.05 level of significance, was applied to determine the statistical difference between the unbound TLR4 structure and IP-bound TLR4 structure. The results show no significant differences in the fluctuation of the amino acid residues of the TLR4-apo and TLR4-IP chain A (*p* = 0.2890) and chain D (*p* = 0.0525).

Although fluctuation differences between Figure 11B,C are not that noticeable, statistical results for chain B (*p* < 0.0001, *p* = 0.0000233886) and chain C (*p* < 0.0001, *p* = 0.0000159098) show that there is a significant difference in the fluctuation of the amino acid residues between the TLR4-apo and TLR4-DahA chains B and C. Additionally, based on Figure 11E–G, the binding of IP showed no significant change in the structure of TLR4 from 0 ns to 10 ns.

**Figure 12:**
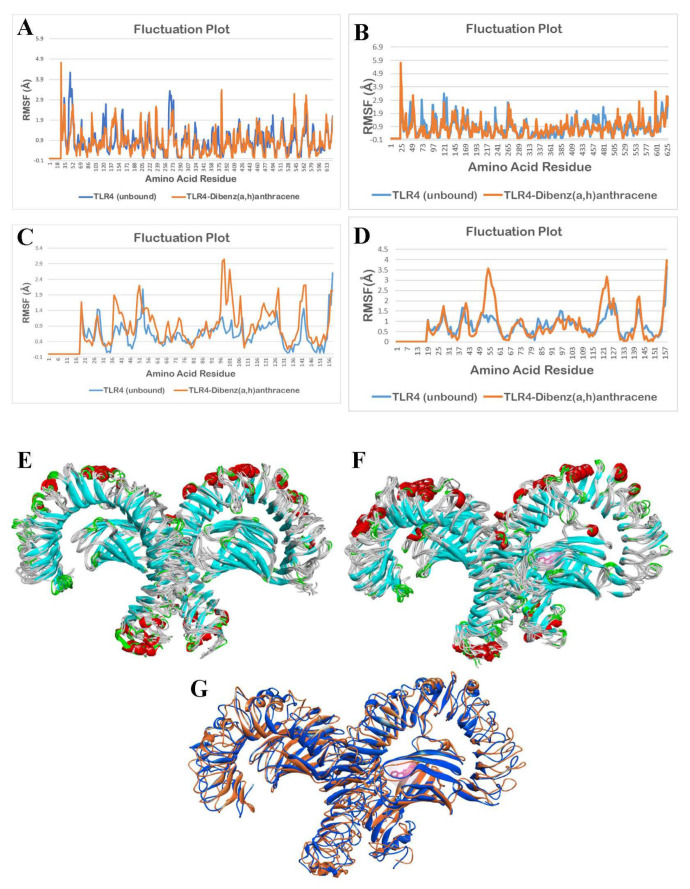
Molecular dynamics simulation of unbound and DahA bound TLR4 structures: (**A**) fluctuation plot of the number of amino acid residues of unbound (blue) and bound structure (orange) of TLR4 chain A, (**B**) fluctuation plot of the number of amino acid residues of unbound (blue) and bound structure (orange) of TLR4 chain B, (**C**) fluctuation plot of the number of amino acid residues of unbound (blue) and bound structure (orange) of TLR4 chain C, (**D**) fluctuation plot of the number of amino acid residues of unbound (blue) and bound structure (orange) of TLR4 chain D, (**E**) superimposition of unbound TLR4 structures from 0 ns to 10 ns, (**F**) superimposition of DahA bound TLR4 structures from 0 ns to 10 ns, and (**G**) superimposition of unbound TLR4 (blue) at 0 ns and DahA (pink) bound TLR4 (orange) at 10 ns.

Fluctuations in the amino acid residues in the unbound (apo/ligand-free) TLR4 and bound structure of TLR4 with DahA during a 10 ns molecular dynamics simulation are shown in Figure 12.

As observed in Figure 12A,B,D, there are minimal fluctuations in the TLR4-apo and TLR4-DahA structures. To determine the statistical difference between the unbound TLR4 structure and the DahA-bound TLR4 structure, a statistical analysis using a two-tailed t-test at a 0.05 level of significance was applied. The results show that there are no significant differences in the fluctuation of the amino acid residues of the TLR4-apo and TLR4-DahA chain A (*p* = 0.1533), chain B (*p* = 0.4273), and chain D (*p* = 0.3354).

As observed in Figure 12C, there is a significant difference between the fluctuations of amino acid residues of TLR4-apo and TLR4-DahA in chain C. The statistical analysis for chain C also shows a significant difference in the fluctuation of the amino acid residues of the TLR4-apo and TLR4-DahA chain C (*p* < 0.0001, *p* = 0.0000047305).

### 3.3. AP Phytocompounds and NF-κB p50 Interaction

In this part of the study, an in silico molecular docking technique was performed using AutoDock Vina to predict the *Andrographis paniculata* (AP) compounds’ conformations in the NF-κB p50 transcription factor. The agonists, polycyclic aromatic hydrocarbons in particulate matter, caused inflammation, as demonstrated by binding with TLR4 activation with higher binding affinities than for LPS. The *Andrographis paniculata* (AP) compounds served as potential antagonists of the NF-κB p50 subunit. At the cellular level, AP compounds could show anti-inflammatory effects by inhibiting the NF-κB pathway activated by PAHs.

#### 3.3.1. Analysis of Docking Scores and Amino Acid Interactions

The NF-κB p50 transcription factor shows that ARG-30 and GLN-306 form hydrogen bonds with dexamethasone, the reference compound. The docking results of the protein-ligand interaction are shown in Table 5. As a positive control for the test, dexamethasone, a drug that people take for anti-inflammation, was used. The structure of the dexamethasone closely also resembles the nine AP compounds, and it has its most stable conformation at a binding energy of −5.4 kcal/mol.

**Figure 13:**
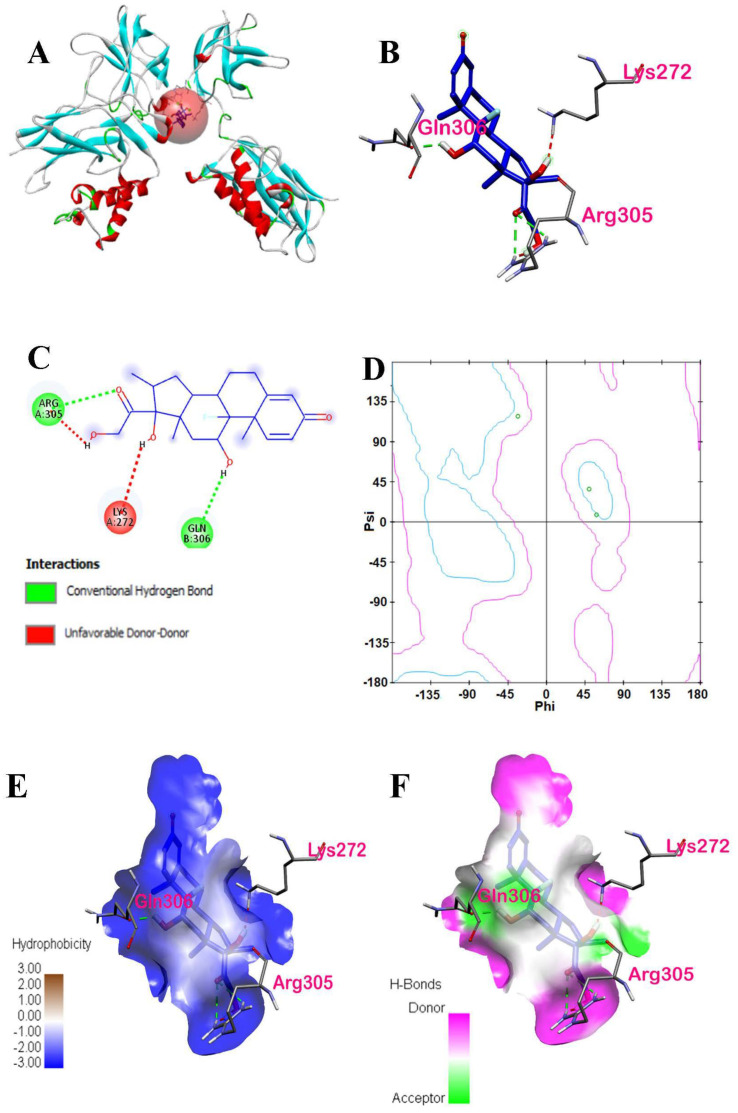
Binding interactions of dexamethasone and p50 subunit in the NF−κB dimer: (**A**) three dimensional visualization of dexamethasone docked at p50 with defined binding site represented by a red sphere, (**B**) three-dimensional interaction of dexamethasone and interacting amino acids of p50, (**C**) two-dimensional diagram visualization of dexamethasone−p50 protein complex, (**D**) Ramachandran plot of ligand-interacting amino acid residues, (**E**) hydrophobic interactions of ligand-interacting amino acid residues, and (**F**) hydrogen bonding of dexamethasone.

A docking study of dexamethasone is shown in Figure 13. The highest docking score is visualized in Figure 13A. Based on the 3D and 2D interactions of dexamethasone with NF-κB p50, three amino acid residues are interacting with the dexamethasone, namely Arg305, Gln306, and Lys272. Moreover, the spatial interaction of dexamethasone with the amino acid residues shows that all these amino acid residues interacted with the ligand by conventional hydrogen bonds, as shown in Figure 13B. However, as shown in Figure 13C, Lys272 formed an unfavorable donor–donor bond. The structural changes upon binding of a few amino acid residues within the binding pocket will be shown later in molecular dynamics simulations.

The Ramachandran plot, as shown in Figure 13D, indicates that the two amino acids (Lys272 in chain A and Gln306 in chain B) are within acceptable or favorable regions, whereas Arg305 in chain A is within the allowed regions. There were no outliers in the plot. It was also revealed that Lys272 is from the β-pleated sheets, whereas Arg305 and Gln306 are from the α-helix structures. In the hydrophobic cloud in Figure 13E, the hydrophobic interactions between the dexamethasone and active site residues were all displayed as conventional hydrogen bonds via the amino acid residues present. Considering carbon-H bond types, Arg305 is an H-donor to dexamethasone whereas dexamethasone is an H donor to Gln306, an H acceptor.

In AutoDock Vina, the docking calculations for the binding energies of each of the nine AP compounds were performed. The calculations were based on the flexibility of the ligands while the NF-κB p50 protein was kept rigid. The compounds from *Andrographis paniculata* were chosen as these were the isolated active compounds from the bioactivity-guided fractionation that showed in vivo anti-inflammatory activities [15]. The results from the molecular docking calculations, which ranged from −5.6 kcal/mol to −4.7 kcal/mol, are shown in increasing order in Table 6. Eight of the phytochemicals of *Andrographis paniculata* had lower binding energies than the reference compound, dexamethasone. Only ergosterol peroxide had higher binding energy than dexamethasone at −5.6 kcal/mol.

The molecular docking results show that ergosterol peroxide had a stronger binding affinity towards the NF-κB p50 protein than dexamethasone. In contrast, 19-O-acetyl-14-deoxy-11,12-didehydroandrographolide had the weakest binding affinity of −4.7 kcal/mol towards the NF-κB p50 protein. Ergosterol peroxide had a stronger binding affinity than dexamethasone.

The 14-deoxy-14,15-dehydroandrographolide and 5-hydroxy-7,8-dimethoxyflavanone were of equal affinities (−5.3 kcal/mol). The former was investigated more closely because a study by Chao, Kuo, & Lin [15] has shown that it possesses the most potent inhibitory activity of in vitro NF-κB transactivation and inflammatory mediators.

**Figure 14:**
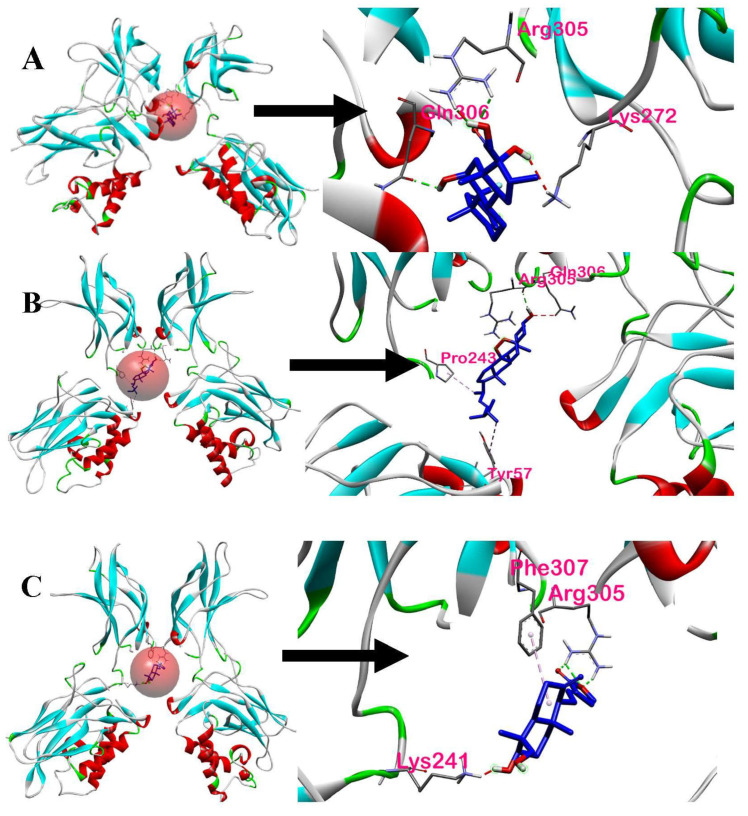
Receptor-ligand interactions: (**A**) dexamethasone with NF-κB p50, (**B**) ergosterol peroxide with NF-κB p50, and (**C**) 14-deoxy-14,15-dehydroandrographolide with NF-κB p50.

The interaction of the AP compounds complexed with the NF-κB p50 protein was visualized in BIOVIA Discovery Studio Visualizer. In Figure 14, the amino acid residues interacting with dexamethasone, ergosterol peroxide, and 14-deoxy-14,15-dehydroandrographolide are defined further by the red sphere and closely shown.

**Figure 15:**
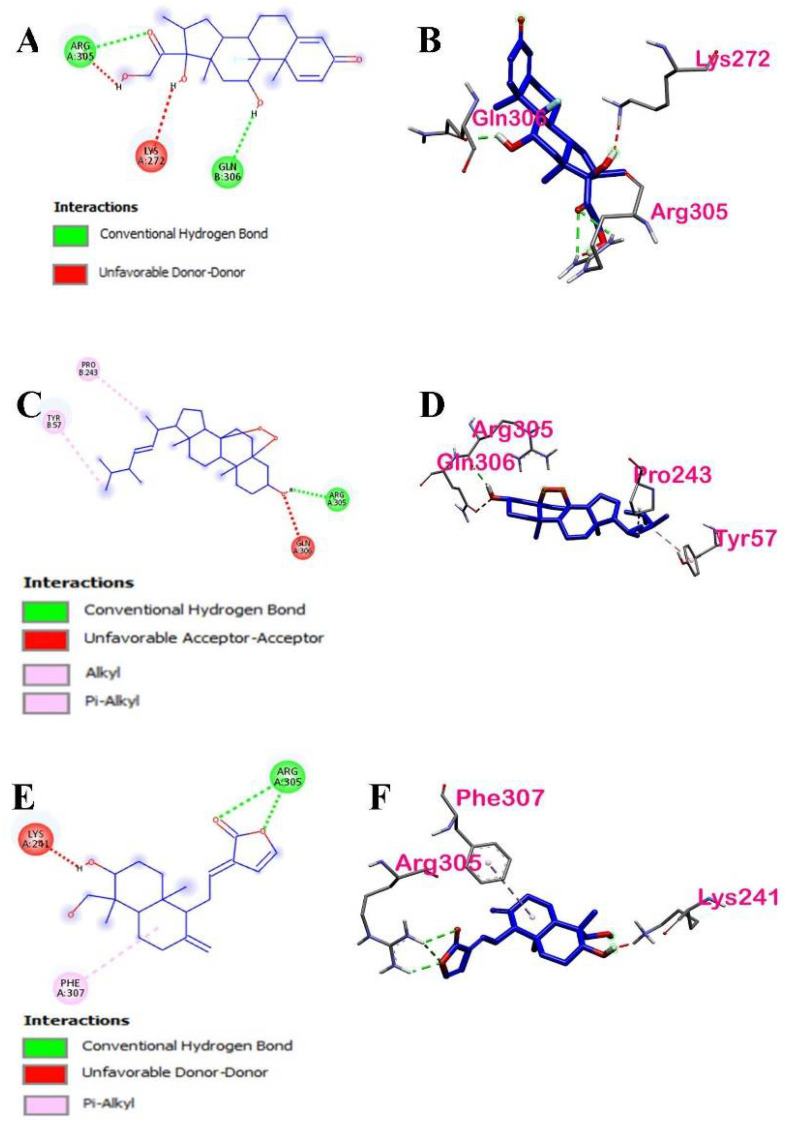
Two- and three-dimensional interactions of dexamethasone and ligands that have highest binding affinities with NF-κB p50: (**A**) 2D interaction of dexamethasone, (**B**) 3D interaction of dexamethasone, (**C**) 2D interaction of ergosterol peroxide, (**D**) 3D interaction of ergosterol peroxide, (**E**) 2D interaction of 14-deoxy-14,15-dehydroandrographolide, and (**F**) 3D interaction of 14-deoxy-14,15-dehydroandrographolide.

Figure 15 shows the interactions between dexamethasone and ligands that have the highest binding affinities with NF-κB p50. Based on the 2D and 3D interactions of the ligands with the highest binding affinities, three amino acids (Arg305, Pro243, and Tyr57) in ergosterol peroxide were found to interact with the NF-κB p50. The amino acids formed various bonds: Arg305 formed a conventional hydrogen bond, Pro243 an alkyl bond, and Tyr57 a pi-alkyl bond, whereas Gln306 formed an unfavorable acceptor–acceptor bond.

Four amino acids (Arg305, Phe307, Lys241, and Lys272) in 14-deoxy-14,15-dehydro-andrographolide were found to interact with NF-κB p50. The ligand of interest and its interacting amino acids displayed various interactions: Arg305 formed a conventional hydrogen bond, Phe307 a pi-alkyl bond, and Lys241 an unfavorable donor–donor bond.

Further details on the interactions between the ligands and NF-κB p50 are shown in Table 7. As discussed earlier, dexamethasone had three interacting amino acids, namely Arg305, Gln306, and Lys22. One of the common amino acids in the binding ligands is Arg305 of the p50 protein which interacted with both ergosterol peroxide and 14-deoxy-14,15-dehydroandrographolide. Arg305 formed a conventional hydrogen bond. On the other hand, the other common acid, Lys272 in 14-deoxy-14,15-dehydroandrographolide, formed an unfavorable donor–donor bond. This could be due to the flexible nature of Lys 272 [30].

#### 3.3.2. Intermolecular Forces of Attraction

**Figure 16:**
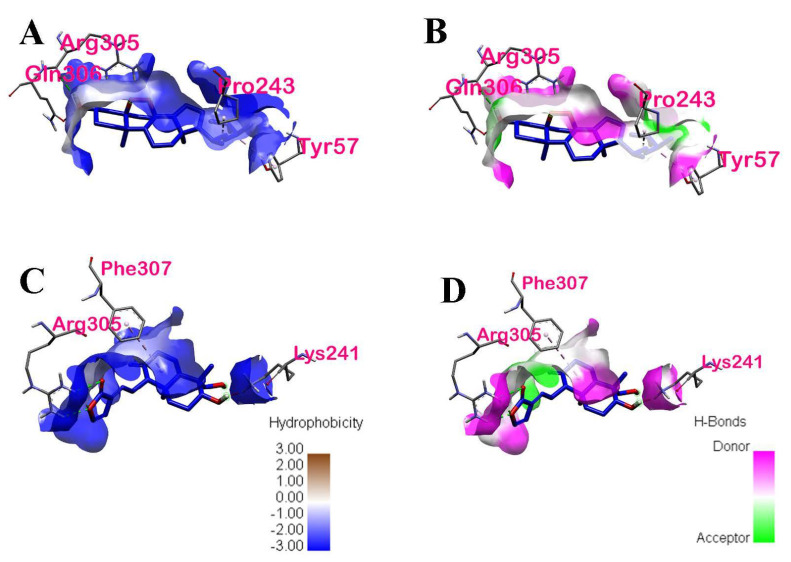
Intermolecular forces of attractions between the top binding ligands and NF−κB p50: (**A**) hydrophobic interactions of ergosterol peroxide, (**B**) hydrogen bonding of ergosterol peroxide, (**C**) hydrophobic interactions of 14-deoxy-14,15-dehydroandrographolide, and (**D**) hydrogen bonding of 14-deoxy-14,15-dehydroandrographolide.

The hydrophobic interactions between the binding ligands with the highest binding energies and their active site residues are shown in Figure 16. The distribution of the hydrophobicity of the two ligands of interest and their respective binding sites are projected onto the surface of NF-κB p50. It can be observed from the hydrophobicity profiles of the ligands of interest that the active pocket amino acids are mostly hydrophilic and that of the aromatic groups are less hydrophilic. The hydrophobic interactions of ergosterol peroxide and the active site residues found in chains A and B of p50 were conventional hydrogen bond, alkyl, and pi-alkyl via Arg305, Pro243, and Tyr57, respectively. The hydrophobic interactions of 14-deoxy-14,15-dehydroandrographolide and the active site residues found in chain A were only conventional hydrogen bonds, and pi-alkyl via Arg 305 and Phe307, respectively.

Both ergosterol and 14-deoxy-14,15-dehydroandrographolide formed hydrogen bonds with p50. The hydrogen bond properties of the binding pocket in the ergosterol peroxide show Arg305 (2.11491 Å) as a conventional H-bond donor to O of the ligand. In contrast, Pro243 (4.50788 Å) and Tyr57 (4.88679 Å) formed hydrophobic interactions with ergosterol peroxide via alkyl and pi-orbital, respectively. In 14-deoxy-14,15-dehydroandrographolide, three bonds of Arg305 (2.73007 Å, 2.69895 Å, and 2.19226 Å) are conventional H-bond donors to O of the ligand, whereas Phe307 (5.19413 Å) formed a hydrophobic interaction with the ligand via pi-alkyl.

From the molecular docking results, it can be speculated that both hydrophobic interactions and hydrogen bonding helped to stabilize the protein–ligand binding. Their binding stability will be further discussed in Section 3.3.4.

#### 3.3.3. Ramachandran Plot

**Figure 17:**
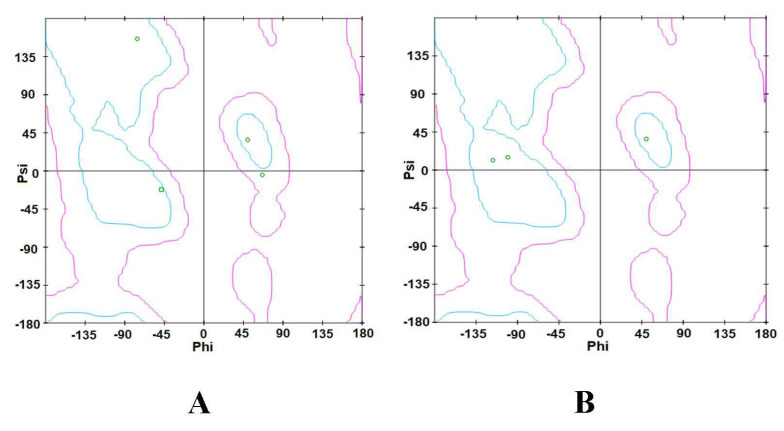
Ramachandran plot of the ligands with highest binding affinities with interacting amino acid residues: (**A**) Ramachandran plot of ergosterol peroxide, and (**B**) Ramachandran plot of 14−deoxy−14,15dehydroandrographolide.

The Ramachandran plots of the binding ligands with the highest binding affinities are shown in Figure 17. The Ramachandran plot of ergosterol peroxide indicates that Tyr57, Pro243, and Gln306 are within the acceptable or favorable regions, whereas Arg305 is within the allowable regions. Arg305 and Gln306 of chain A of p50 are α-helix structures, whereas Tyr57 and Pro243 of chain B of p50 are β-pleated sheets. In the Ramachandran plot of 14-deoxy-14,15-dehydroandrographolide, the three amino acids (Arg305, Phe307, and Lys241) found in chain A of the p50 subunit are within the allowed regions. Arg305 is an α-helix structure, whereas Phe307 and Lys241 are β-pleated sheets. These secondary structures are local structures stabilized by hydrogen bonds.

#### 3.3.4. Molecular Dynamics Simulation

**Figure 18:**
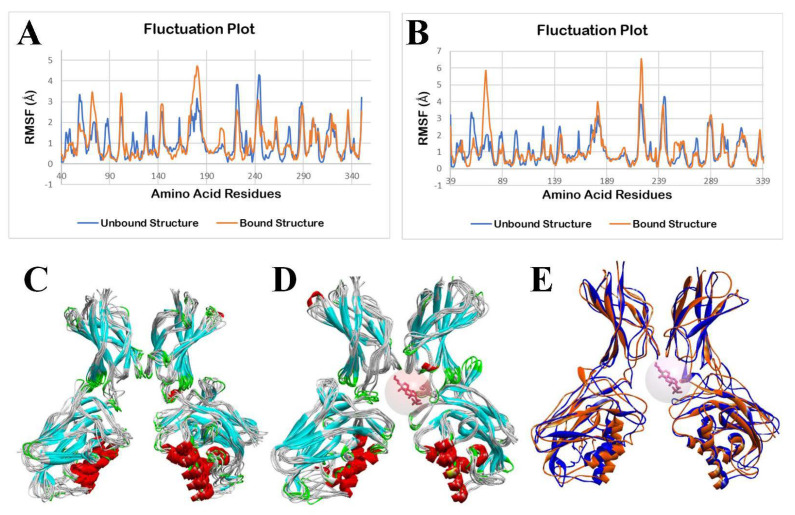
Molecular dynamics simulation of unbound and ergosterol peroxide bound NF−κB p50 structures: (**A**) fluctuation plot of the number of amino acid residues of unbound (blue) and bound structure (orange) of NF−κB p50 chain A, (**B**) fluctuation plot of the number of amino acid residues of unbound (blue) and bound structure (orange) of NF−κB p50 chain B, (**C**) superimposition of unbound NF−κB p50 structures from 0 ns to 10 ns, (**D**) superimposition of ergosterol peroxide bound NF−κB p50 structures from 0 ns to 10 ns, and (**E**) superimposition of unbound NF−κB p50 (blue) at 0 ns and ergosterol peroxide (pink) bound NF−κB p50 (orange) at 10 ns.

The molecular dynamics simulation (MDS) was performed using the CABS-flex 2.0 web server, applying the default parameters. Fluctuations in the amino acid residues in the unbound (apo/ligand-free) NF-κB p50 and bound structure of NF-κB p50 with ergosterol peroxide during a 10 ns molecular dynamics simulation are shown in Figure 18. There are only two chains in the NF-κB p50, A and B. The root mean square fluctuation (RMSF) of amino acids of the p50-apo complex is shown in blue, and the p50-ligand complex is shown in orange in the fluctuation plot. RMSF characterizes the residual flexibility and local changes in the protein chain.

As shown in Figure 18A,B, it can be observed that there are minimal fluctuations in the p50-apo and p50-ergosterol peroxide structure. An analysis was performed to determine the statistical differences between the unbound NF-κB p50 structure and ergosterol peroxide-bound NF-κB p50 structure. In the two-tailed t-tests, there are no significant differences in the fluctuation of the amino acid residues of the p50-apo and p50-ergosterol peroxide chain A (*p* = 0.9966) and chain B (*p* = 0.8286).

**Figure 19:**
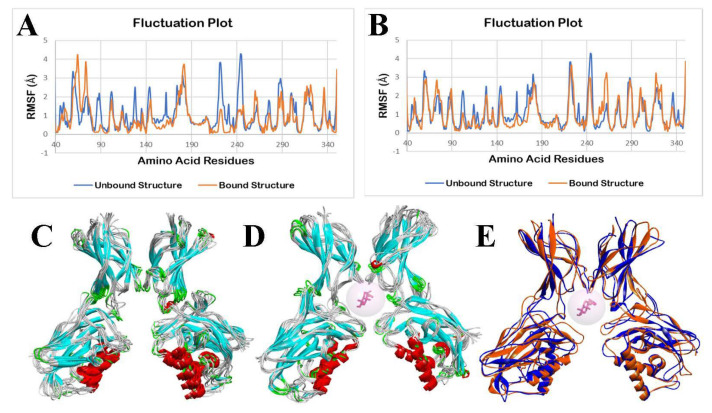
Molecular dynamics simulation of unbound and 14−deoxy−14,15−dehydroandrographolide bound NF−κB p50 structures: (**A**) fluctuation plot of the number of amino acid residues of unbound (blue) and bound structure (orange) of NF−κB p50 chain A, (**B**) fluctuation plot of the number of amino acid residues of unbound (blue) and bound structure (orange) of NF−κB p50 chain B, (**C**) superimposition of unbound NF−κB p50 structures from 0 ns to 10 ns, (**D**) superimposition of 14−deoxy14,15−dehydroandrographolide bound NF−κB p50 structures from 0 ns to 10 ns, and (**E**) superimposition of unbound NF-κB p50 at 0 ns and 14−deoxy−14,15−dehydroandrographolide bound NF−κB p50 at 10 ns.

Fluctuations in the amino acids in the p50-apo and p50-14-deoxy-14,15-dehydroandrographolide complex during a 10 ns molecular dynamic simulation are shown in Figure 19A,B. From Figure 19A, it can be observed that there are some significant fluctuations in the p50-14-deoxy-14,15-dehydroandrographolide chain A complex, whereas there are only minimal fluctuations in chain B. The superimposed p50-14-deoxy-14,15-dehydroandrographolide structures during a run from 0 ns to 10 ns showed observable changes in one part of its dimer.

Statistical analysis of a two-tailed t-test showed that there is a significant difference in chain A (*p* = 0.0038), whereas there is no significant difference in the fluctuations of amino acid residues in chain B (*p* = 0.3147).

## 4. Discussion

The binding energies of all the seven (7) PAHs to TLR4 were determined in AutoDock Vina. For further discussions, PAHs with the highest binding affinities were exhaustively analyzed. The PAHs with lower binding energies were preferred over PAHs with higher binding energies. Lower binding energy is preferred because the lower the binding energy is, the higher the binding affinity will be, thus resulting in a stronger and more stable complex between the ligand and the protein. Based on Table 1, LPS has a higher binding energy, and hence, a lower binding affinity than the 7 PAHs. These results suggest that IP, DahA, BkF, BaP, BbF, Chrysene, and BaA can potentially induce inflammation via the TLR4 pathway. Among the 16 carcinogenic PAHs investigated by Sreeja et al. [31], chrysene, BaA, BbF, BkF, DahA, and BaP were also included to find the association against TLR4. Results from Sreeja et al. showed higher binding energy values (e.g., BaA: −7.14 kcal/mol, BbF: −7.695 kcal/mol, BkF: −7.50 kcal/mol, chrysene: −7.02 kcal/mol, IP = −8.25 kcal/mol, etc.) compared with the results of this study (Table 2). This discrepancy in binding energy values may be attributed to the difference in the docking tool used between this study (AutoDock Vina 1.2.0) and by Sreeja et al. (AutoDock 1.5).

Similar to LPS, the results showed that Chain D amino acids of TLR4 (LEU61, ILE32, ILE52, VAL48, and PHE119) also interacted with DahA and IP. According to Cuadrado et al. [32], LPS causes the expression of pro-inflammatory cytokines by binding to TLR4. This consequently causes the activation of NF-κB so that the expression of pro-inflammatory mediators and enzymes is regulated [32]. Given this information, docking of DahA and IP to TLR4 can induce the expression of pro-inflammatory cytokines and activate the canonical NF-κB pathway.

Saturated fatty acids (SFA) in the lipid A moiety of LPS are also essential in activating the TLR4 signaling pathways [33]. This suggests that TLR-mediated target gene expressions can induce cellular inflammation. This can also explain LPS’s ability to induce pro-inflammatory responses through the TLR4 pathway. According to Ye et al. [34], branched-chain amino acids (BCAAs) and fatty acids significantly affect mitochondrial biogenesis, energy metabolism, and upregulation of inflammatory signals. The activation of mTORC1 and the upregulation of the NF-κB signaling pathway, through the supplementation of BCAAs, can induce the release of pro-inflammatory cytokines in human endothelial cells and peripheral blood mononuclear cells. Furthermore, aside from BCAAs and fatty acid chains [34], aromatic compounds are also known to induce inflammation [35].

The molecular structure of PAHs consists of two or more fused aromatic rings. Thus, they may also be a potential source of inflammation. As seen in Figure 9A,C, DahA and IP are hydrophobic. Due to their hydrophobicity, DahA and IP are small enough to be able diffuse or interact with the proteins of the amphipathic cell membrane and enter the cell [36]. PAHs are usually found in mixtures in ambient air, but they exist as mixtures of many diverse types, thus making analyses of individual PAHs extremely difficult [37]. Furthermore, a mixture of PAHs is known to cause irritation and inflammation to both animal and human skin. One example of a known PAH skin irritant is benzo(a)pyrene (BaP). However, although individual PAHs such as BaP can have adverse health effects, a mixture of PAHs is reported to be more toxic than BaP alone [18].

IP and DahA have the lowest binding energies, which suggests that IP and DahA formed the strongest ligand–protein complexes among the seven PAHs. Furthermore, since PAHs usually occur in mixtures, toxic equivalent factors (TEFs), found in Table 3, can be used to express a PAH’s toxicity by representing it as a single number equivalent to the concentration of the most toxic and carcinogenic PAH [30]. As shown in Table 3, IP and BkF each have a toxicity equivalent factor of 0.1. Therefore, IP and BkF’s toxicity are one-tenth that of BaP. On the other hand, since DahA has a toxicity equivalent factor equal to 1, DahA and BaP have the same toxicity levels. Thus, despite having a stronger affinity for IP, DahA may be more toxic to the human body due to a higher TEF value and can, therefore, be used as an inflammatory inducer while using AP compounds as the inflammatory inhibitor in future studies.

Based on statistical analysis, no significant difference was found in the fluctuations between the amino acids of TLR4-apo and TLR4-IP structures of chains A (*p* = 0.2890) and D (*p* = 0.0525), suggesting that the protein integrity of TLR4 is retained and it thus induces inflammation through the TLR4 pathway. In contrast, a significant difference was observed between the fluctuation of the amino acid residues between the TLR4-apo and TLR4-IP chains B (*p* < 0.0001) and C (*p* < 0.0001). This suggests that the binding of IP to chains B and C of TLR4 cannot create a stable complex and it ceases to perform its natural function. However, since the binding site and amino acid interaction between IP and TLR4 only occurs in the chain D of TLR4, the binding of IP to TLR4 will not cause TLR4 to lose its natural function. This is consistent with the results shown in Figure 10E–G because IP showed no notable change in the structure of TLR4 from 0 ns to 10 ns. This suggests that the human TLR4 is still stable after the docking of IP into its respective binding sites. For DahA, there was no significant difference (*p* > 0.05) in the fluctuations between the amino acids of TLR4-apo and TLR4-DahA structures of chains A (*p* = 0.1533), B (*p* = 0.4273), and D (*p* = 0.3354). This suggests that the binding of DahA to chains A, B, or D of TLR4 will not cause TLR4 to lose its protein integrity. Therefore, DahA also has the potential to induce inflammation through the TLR4 pathway without compromising the TLR4 protein structure.

In contrast, a significant difference (*p* < 0.0001) was observed in the fluctuations between the amino acid residues of TLR4-apo and chain C TLR4-DahA. This suggests that the binding of DahA to chain C of TLR4 cannot create a stable complex and cause TLR4 to lose its protein integrity. However, since the results showed that amino acid interaction between DahA and TLR4 only occurs in the chain D of TLR4 (Table 2), the binding of DahA to TLR4 does not disrupt its natural protein functions. This is consistent with the results shown in Figure 11E–G because DahA showed no notable change in the structure of TLR4 from 0 ns to 10 ns. Moreover, previous results showed that LPS interacts with chain D of TLR4, which suggests that DahA-TLR4 and IP-TLR4 complexes may potentially induce inflammation through the TLR4 pathway in the same way that LPS induces inflammation.

The prolonged use of anti-inflammatory drugs could initiate adverse effects. Thus, the anti-inflammatory capacity of the AP phytocompounds is targeted as a new alternative to these drugs. Most classes of drugs treating human inflammatory diseases focus on inhibiting NF-κB activation.

The *in silico* results suggest that the investigated phytocompound (ergosterol peroxide) of AP showed better inhibitory activity against the p50 subunit in the NF-κB pathway (which plays a vital role in the pathogenesis of inflammation) than the standard dexamethasone and the other investigated phytocompound (14-deoxy-14,15-dehydroandrographolide). These phytocompounds were subjected to molecular docking against the p50 protein. This conclusion is evident from the high binding energies of the docked phytocompounds to NF-κB p50 transcription factors which were −5.6 kcal/mol for ergosterol peroxide and −5.3 kcal/mol for 14-deoxy-14,15-dehydroandrographolide. 

There were three amino acids in the interactions of dexamethasone with the NF-κB p50 protein, namely Arg305, Gln306, and Lys272. The two-dimensional diagram showed that only Lys272 formed an unfavorable donor–donor bond, whereas Arg305 and Gln306 formed conventional hydrogen bonds with dexamethasone. According to Berry et al. [22], lysine is more flexible than arginine and glutamine side chains upon ligand binding. In the Ramachandran plot of ligand-interacting amino acid residues of dexamethasone, lysine rarely contributes to binding in their native form because the basicity of the side-chain renders substituents to be positively charged at physiological pH. Thus, it is used to improve the receptor’s water solubility [38]. All three active amino acid residues surrounding the ligand are hydrophilic, as shown in Figure 13E. 

Furthermore, phytocompounds with the highest binding affinities were analyzed. It was found that the molecular interactions of ergosterol peroxide and 14-deoxy-14,15-dehydroandrographolide had the lowest binding energies, as shown in Table 6. Since ergosterol peroxide has a stronger binding affinity than dexamethasone, it has the potential to inhibit the p50 subunit in the NF-κB pathway and thus reduce inflammation. Furthermore, the root mean square deviation (RMSD) for all the best binding affinities of the reference inhibitor dexamethasone and the nine phytochemicals of AP were measured to be 0.00 angstrom (Ǻ), which indicates efficacious docking. This value is the parameter that validates the accuracy of the molecular docking. At 0 Ǻ, the divergence between the coordinates of the crystallized complex of the simulated complex is determined. Hence, the RMSD value of 0 Ǻ is evidence of the best docking pose in the binding site (i.e., lowest binding energy, higher ranking, strongest binding affinity). 

Anti-inflammatory compounds inhibit inflammation processes on the canonical NF-κB pathway, which is responsible for forming inflammasomes and expressing pro-inflammatory target genes [21,39,40,41]. In this pathway, the bioactive compounds from *Andrographis paniculata* are studied using docking analysis of the ligands with the highest binding energies and the NF-κB p50 transcription factor as the target protein. The possible inhibition of the target protein is beneficial because it, in turn, inhibits the expression of the NF-κB targeted genes. Ergosterol peroxide is a sterol found in *Andrographis paniculata* and has been reported to have various pharmacological properties such as anti-microbial, anti-tumor, and anti-inflammatory effects [41]. This phytocompound from AP has been reported to act on molecular targets such as NF-κB. It has also been reported that ergosterol peroxide inhibits RIG-I in influenza A virus-induced inflammation which results in the downregulation of pro-inflammatory expression. Although 14-deoxy-14,15-dehydroandrographolide is a diterpenoid, one of the less abundant compounds from *A. paniculata* has anti-inflammatory properties [15]. From the screening study of the eight analogs of andrographolide conducted by Tan et al. [6], 14-deoxy-14,15-dehydroandrographolide is one of the phytocompounds that is found to have more potent inhibitory effects on NF-κB transactivation, and TNF-α, IL-6, MIP-2 and NO expressions compared with andrographolide, a significant constituent of *A. paniculata*. Moreover, its structure has an α-alkylidene-β,γ-unsaturated-γ-lactone, which has been reported to show a superior inhibitory impact against NF-κB activity [15].

An MDS of 10 ns was performed on the investigated phytocompounds, which had the lowest docking energy conformation with the target protein. The root mean square fluctuations characterized the residual flexibility and local changes along the NF-κB p50 protein chains. The docked phytocompounds formed stable complexes, except for the protein chain A of 14-deoxy-14,15-dehydroandrographolide, which showed significantly higher fluctuations with the NF-κB p50 transcription factor. Minimal fluctuations were insignificant, and thus, the stability of the p50-apo and p50-ergosterol peroxide chain A and chain B complex are maintained throughout the simulation based on the generated RMSF plots (chain A, *p* = 0.9966; chain B *p* = 0.8286). Post molecular dynamic simulations revealed minimal changes in the general structure when superimposed with the pre-MDS p50-apo. This numerical data supports the minimal fluctuations observed in the ergosterol peroxide bound NF-κB p50 (orange) in the superimposed models, as shown in Figure 18C–E. It may be concluded that the NF-κB p50 structure is rigid when ergosterol peroxide is bound. Therefore, this suggests that ergosterol peroxide can form a stable complex with NF-κB p50 and an excellent potential steady binding that could serve as a candidate anti-inflammatory drug. Minimal fluctuations were observed in amino acid residues in the p50 and 14-deoxy14,15-dehydroandrographolide chain B complex (*p* = 0.3147) compared with the significant difference in the chain A complex based on the generated RMSF plot *p* = 0.0038). This result suggests that the 14-deoxy-14,15-dehydroandrographolide chain A complexed with NF-κB p50 did not create stable binding, and this was validated by the RMSF output. However, 14-deoxy-14,15-dehydroandrographolide chain B showed an excellent potential steady binding with the NF-κB p50 structure, creating a stable complex, and thus, it could be a viable candidate for use as an anti-inflammatory drug.

Based on an extensive search of the nine AP phytocompounds in Table 6, it was determined that 5-hydroxy-7,8-dimethoxyflavanone has not been reported to have an anti-inflammatory effect. The phytocompound has a −5.3 kcal/mol binding energy similar to 14-deoxy-14,15-dehydroandrographolide. Its moderate binding affinity against the target protein is worth investigating in in vitro experiments in the future. Other AP phytocompounds, 5-hydroxy-7,8-dimethoxyflavone, and 19-O-acetyl-14-deoxy-11,12-didehydroandrographolide significantly inhibited the transcriptional activity of NF-κB in LPS/IFN-γ stimulated RAW 264.7 macrophages [15]. The latter is a new compound that is also worth investigating in in vitro studies, and the former, a flavone, has been observed to have anti-inflammatory, antioxidant, and antiproliferative activity [42]. In addition, 14-deoxy-11,12-didehydroandrographolide is reported to be a safer analog of andrographolide for the potential treatment of asthma [43] and significantly inhibited pro-inflammatory cytokines/chemokines expression in the H5N1 virus pathogenesis [44]. In vivo and in silico studies on andrographolide revealed a reduction of p65 phosphorylation level in the lungs of OVA-treated mice and suppression of LPS-induced NO, IL-6, and TNF-α production in macrophages and cytokines [45]. Stigmasterol has been shown to strongly suppress the expression of pro-inflammatory mediators (TNF-α, IL-6, IL-1β, iNOS, and COX-2) and increases the expression of anti-inflammatory cytokine (IL-10) by down-regulation of NF-κB p65 in CIA rats [46]. Furthermore, β-sitosterol has shown anti-inflammatory activity by attenuating the phosphorylation of NF-κB p65 in human aortic endothelial cells [47]. 

An IKK phosphorylation and covalent binding of the ligands to the p50 protein indicates a cellular anti-inflammatory mechanism [48]. Moreover, the anti-inflammatory mechanism, inhibition of NF-κB activity, is yet to be elucidated for the other AP phytocompounds. These ligands of interest, ergosterol peroxide and 14-deoxy-14,15-dehydroandrographolide, with high binding affinities from AP, remained inside the protein’s binding site throughout the simulation. These results suggest that these phytocompounds of AP can be responsible for the potential anti-inflammatory effects of AP based on the in silico screening. The AP phytocompounds as a therapeutic antagonist to the NF-κB p50 protein are worthy of identification for future in vitro and in vivo studies.

## 5. Conclusions

An active TLR4 can subsequently activate the NF-κB pathway. This transmembrane protein causes the downstream expression of various pro-inflammatory cytokines. A docking analysis was performed, and the binding energies were compared. Indeno(1,2,3-cd)pyrene and dibenz(a,h)anthracene are two of the most promising polycyclic aromatic hydrocarbons investigated in the study. Analysis of these two PAHs showed higher binding affinities than the reference pro-inflammatory compound LPS. The PAHs mimicked the LPS by binding to TLR4, which maintains the activation of the NF-κB pathway and upregulates pro-inflammatory cytokines. The analysis showed that the amino acid interactions between the ligands and the human TLR4 were dominated by pi-system interactions. Further analysis revealed that the active pocket amino acid residues surrounding the ligands of interest are strongly hydrophobic. Moreover, only the stable binding between IP and TLR4 structures chain A and chain D showed significant pro-inflammatory activity, as indicated by the minimal root mean square fluctuations between the apo and bound structure. Dibenz(a,h)anthracene can stably bind to the TLR4 structure, as shown by the minimal root mean square fluctuations between the apo and bound structures of chain A, chain B, and chain D of TLR4.

Ergosterol peroxide showed potent inhibitory effects on NF-κB transactivation. As revealed by docking analysis, this phytocompound was revealed to have a higher binding affinity than the reference inhibitor dexamethasone. Further analysis showed that the interactions of the two AP phytochemicals with the NF-κB p50 are conventional hydrogen bonds, alkyl, and pi-alkyl. The results also showed that the ligands’ active pocket amino acids are hydrophilic. The NF-κB p50 complex with ergosterol peroxide had stable conformation as minimal fluctuations were observed between the protein’s apo and the bound structures of chain A and chain B. Only 14-deoxy-14,15-dehydroandrographolide and chain B of NF-κB p50 formed stable binding, evidenced by the minimal root mean square fluctuations between the apo and bound structure. Through an in silico study, ergosterol peroxide was demonstrated to be a suitable inhibitor of the NF-κB p50 protein.

The expression of pro-inflammatory genes induced by PAHs may be inhibited by *A. paniculata* compounds by targeting the homodimer NF-κB p50. Therefore, the researchers theorize that indeno(1,2,3-cd)pyrene (IP)- and dibenz(a,h)anthracene-induced inflammations are potentially inhibited by *A. paniculata* compounds, particularly ergosterol peroxide and 14-deoxy-14,15-dehydroandrographolide. Confirmatory in vitro and in vivo studies on the identified phytocompounds are recommended for future studies to determine their ameliorative and cytotoxic properties.

## Figures and Tables

**Table 1:** Binding energy of reference compound lipopolysaccharide with the human Toll-like receptor 4.

Ligand Reference	Binding Energy (kcal/mol)	Interacting Amino Acids	Interaction	Type	Location of Interaction
LPS	−4.1	LYS122	Attractive charge	Hydrogen Bond	Chain D of TLR4
		ARG264	Conventional hydrogen bond	Hydrogen Bond	Chain B of TLR4
		LYS362	Conventional hydrogen bond	Hydrogen Bond	Chain B of TLR4
		ARG90	Conventional hydrogen bond	Hydrogen Bond	Chain D of TLR4
		TYR102	Conventional hydrogen bond	Hydrogen Bond	Chain D of TLR4
		LYS122	Conventional hydrogen bond	Hydrogen Bond	Chain D of TLR4
		GLU439	Conventional hydrogen bond	Hydrogen Bond	Chain A of TLR4
		LYS362	Alkyl	Hydrophobic	Chain B of TLR4
		VAL48	Alkyl	Hydrophobic	Chain D of TLR4
		ILE52	Alkyl	Hydrophobic	Chain D of TLR4
		LEU61	Alkyl	Hydrophobic	Chain D of TLR4
		LEU78	Alkyl	Hydrophobic	Chain D of TLR4
		ILE124	Alkyl	Hydrophobic	Chain D of TLR4
		CYS133	Alkyl	Hydrophobic	Chain D of TLR4
		VAL135	Alkyl	Hydrophobic	Chain D of TLR4
		ILE32	Alkyl	Hydrophobic	Chain D of TLR4
		ILE94	Alkyl	Hydrophobic	Chain D of TLR4
		PHE119	Pi-alkyl	Hydrophobic	Chain D of TLR4

**Table 2:** Interactions between the highest binding affinity ligands with the human Toll-like receptor 4 (TLR4).

Ligand	Binding Energy (kcal/mol)	Interacting Amino Acids	Interaction	Type	Location of Interaction	Common Amino Acid LPS and Ligands Interacted with
IP	−10	ILE52, PHE121, PHE151, LEU61, ILE32, VAL48	Hydrophobic	Pi-sigma and Pi-alkyl, Pi-Pi stacked, Pi-Pi T-shaped, Pi-alkyl, Pi-alkyl, Pi-alkyl	Chain D of TLR4	4: Val48, ILE52, ILE32 and LEU61
DahA	−9.2	PHE121, PHE119	Hydrophobic	Pi-Pi stacked, Pi-Pi stacked	Chain D of TLR4	1: PHE119
BkF	−8.9	PHE119, PHE121, ILE52, LEU61	Hydrophobic	Pi-Pi stacked, Pi-Pi stacked, Pi-Alkyl, Pi-Alkyl respectively	Chain D of TLR-4	3: PHE119, ILE52, and LEU61
BaP	−8.9	PHE119, PHE121, ILE52, LEU61	Hydrophobic	Pi-Pi stacked, Pi-Pi stacked, Pi-Alkyl, Pi-Alkyl respectively	Chain D of TLR-4	3: PHE119, ILE52, and LEU61

**Table 3:** Binding energy results of the 7 PAHs using AutoDock Vina; toxic equivalent factors (TEF); and carcinogenic classifications based on the EPA.

Ligand	PubChem ID	Binding Energy (kcal/mol)	TEF	Carcinogenic Classification
IP	9131	−10	0.1	Probable human carcinogen
DahA	5889	−9.2	1	Probable human carcinogen
BkF	9158	−8.9	0.1	Probable human carcinogen
BaP	2336	−8.9	1	Probable human carcinogen
BbF	9153	−8.8	0.1	Probable human carcinogen
Chrysene	9171	−8.2	0.01	Probable human carcinogen
BaA	5954	−8.1	0.1	Probable human carcinogen

**Table 4:** Distance of interaction between ligand and TLR4 amino acids.

Ligand	Amino Acid	Interaction	Type	Distance
IP	ILE52	Hydrophobic	Pi-sigma, Pi-sigma, Pi-sigma, Pi-Alkyl	3.3891, 3.89425, 3.44148, 4.62034
	PHE121	Hydrophobic	Pi-Pi stacked, Pi-Pi stacked	4.14965, 4.64929
	PHE151	Hydrophobic	Pi-Pi T-shaped	5.84498
	LEU61	Hydrophobic	Pi-Alkyl	5.18222
	ILE32	Hydrophobic	Pi-Alkyl	5.47123
	VAL48	Hydrophobic	Pi-Alkyl	5.16885
DahA	PHE119	Hydrophobic	Pi-Pi stacked, Pi-Pi stacked, Pi-Pi stacked	5.52106, 4.47965, 5.42439
	PHE121	Hydrophobic	Pi-Pi stacked, Pi-Pi stacked, Pi-Pi stacked	5.11694, 3.86586, 3.74824

**Table 5:** Binding energy of reference compound dexamethasone with the p50 subunit in the NF-κB dimer.

Ligand Reference	Binding Energy (kcal/mol)	Interacting Amino Acids	Interaction	Type	Location of Interaction
Dexamethasone	−5.4	ARG305	Conventional Hydrogen Bond	Hydrogen Bond	Chain A of p50
		GLN306	Conventional Hydrogen Bond	Hydrogen Bond	Chain B of p50
		LYS272	Conventional Hydrogen Bond	Hydrogen Bond	Chain A of p50

**Table 6:** Binding energy results of dexamethasone and phytochemicals of *Andrographis paniculata* with NF-κB p50.

AP Phytochemical	PubChem ID	Binding Energy (kcal/mol)
Dexamethasone *	5743	−5.4
ergosterol peroxide	5351516	−5.6
14-deoxy-14,15-dehydroandrographolide	5351516	−5.3
5-hydroxy-7,8-dimethoxyflavanone	13963770	−5.3
5-hydroxy-7,8-dimethoxyflavone	188316	−5.2
14-deoxy-11,12-didehydroandrographolide	5708351	−5.2
andrographolide	5318517	−5.2
stigmasterol	5280794	−5.1
β-sitosterol	222284	−5.0
19-O-acetyl-14-deoxy-11,12-didehydroandrographolide	46179874	−4.7

* Positive control.

**Table 7:** Interactions between the binding ligands with lowest binding energies and NF-κB p50.

Ligand	Binding Energy (kcal/mol)	Interacting Amino Acids	Interactions	Type	Location of Interaction	Common Amino Acid Dexamethasone and Ligands Interacted with
Ergosterol peroxide	−5.6	ARG305, PRO243, TYR57	Hydrogen Bond, Hydrophobic	Conventional Hydrogen Bond, Alkyl, Pi-Alkyl	Chain A and Chain B of p50	1: ARG305
14-deoxy-14,15-dehydroandrographolide	−5.3	ARG305, PHE307, LYS241, LYS272	Hydrogen Bond, Hydrophobic	Conventional Hydrogen Bond, Pi-Alkyl	Chain A of p50	2: ARG305, LYS272

## Data Availability

Not applicable.
